# Field- and time-normalization of data with many zeros: an empirical analysis using citation and Twitter data

**DOI:** 10.1007/s11192-018-2771-1

**Published:** 2018-05-19

**Authors:** Robin Haunschild, Lutz Bornmann

**Affiliations:** 1 Max Planck Institute for Solid State Research, Heisenbergstr. 1, 70569 Stuttgart, Germany; 2 Division for Science and Innovation Studies, Administrative Headquarters of the Max Planck Society, Hofgartenstr. 8, 80539 Munich, Germany

**Keywords:** Data with many zeros, Citation counts, Altmetrics, Twitter, Mantel–Haenszel quotient (MHq), Equalized mean-based normalized proportion cited (EMNPC), Mean-based normalized proportion cited (MNPC)

## Abstract

Thelwall (J Informetr 11(1):128–151, 2017a. 10.1016/j.joi.2016.12.002; Web indicators for research evaluation: a practical guide. Morgan and Claypool, London, 2017b) proposed a new family of field- and time-normalized indicators, which is intended for sparse data. These indicators are based on units of analysis (e.g., institutions) rather than on the paper level. They compare the proportion of mentioned papers (e.g., on Twitter) of a unit with the proportion of mentioned papers in the corresponding fields and publication years. We propose a new indicator (Mantel–Haenszel quotient, MHq) for the indicator family. The MHq is rooted in the Mantel–Haenszel (MH) analysis. This analysis is an established method, which can be used to pool the data from several 2 × 2 cross tables based on different subgroups. We investigate using citations and assessments by peers whether the indicator family can distinguish between quality levels defined by the assessments of peers. Thus, we test the convergent validity. We find that the MHq is able to distinguish between quality levels in most cases while other indicators of the family are not. Since our study approves the MHq as a convergent valid indicator, we apply the MHq to four different Twitter groups as defined by the company Altmetric. Our results show that there is a weak relationship between the Twitter counts of all four Twitter groups and scientific quality, much weaker than between citations and scientific quality. Therefore, our results discourage the use of Twitter counts in research evaluation.

## Introduction

Alternative metrics (altmetrics) is a new fast-moving area in scientometrics (Galloway et al. 2013). Initially, altmetrics—a collection of many web-based indicators—have been proposed as a supplement to traditional bibliometric indicators. They measure attention related to research papers on internet platforms. The core of altmetrics is gathered from social media platforms, but mentions in mainstream media or in policy documents also belong to the umbrella term altmetrics (National Information Standards Organization 2016; Work et al. 2015). According to Haustein (2016), sources of altmetrics can be grouped into (i) social networks, (ii) social bookmarks and online reference management, (iii) social data (e.g., data sets, software, presentations), (iv) blogs, (v) microblogs, (vi) wikis, and (vii) recommendations, ratings, and reviews.

Recently, some indicators based on altmetrics have been proposed which are normalized with respect to the scientific field and publication year. These indicators were developed because studies have shown that altmetrics are—similar to bibliometric data—field- and time-dependent (see, e.g., Bornmann 2014). Some fields are more relevant to the general public or a broader audience than other fields (Haustein et al. 2014). The Mean Normalized Reader Score (MNRS) was introduced by Haunschild and Bornmann (2016) for normalization of data from social bookmarks and online reference management platforms (with a special emphasis on Mendeley readers) (see also Fairclough and Thelwall 2015). The Mean Discipline Normalized Reader Score (MDNRS) was tailored specifically to Mendeley by Bornmann and Haunschild (2016b). The MDNRS uses Mendeley disciplines for field normalization. The employed normalization procedures rely on average value calculations across scientific fields and publication years as expected values.

However, normalization procedures based on averages (and percentiles) of individual papers are problematic for data sets with many zeros because averages can get close to zero and only few percentile ranks are occupied (Haunschild et al. 2016). The overview of Work et al. (2015) on studies investigating the coverage of papers on social media platforms show that less than 5% of the analyzed papers were mentioned on many platforms (e.g., Blogs or Wikipedia). Erdt et al.(2016) reported similar findings in their meta-analysis. They found that former empirical studies dealing with the coverage of altmetrics show that about half of the platforms are at or below 5%; except for three (out of eleven) where the coverage is below 10%.

Bornmann and Haunschild (2016a) propose the Twitter Percentile (TP)—a field- and time-normalized indicator for Twitter data. Bornmann and Haunschild (2016a) circumvent the problem of Twitter data with many zeros by including in the TP calculation only journals with at least 80% of the papers having at least 1 tweet each. However, this procedure leads to the exclusion of many journals from the TP procedure.

Very recently, Thelwall (2017a, b) proposed a new family of field- and time-normalized indicators. These indicators are based on units of analysis (e.g., a researcher or institution) rather than on single papers. They compare the proportion of mentioned papers (e.g., on Twitter) of a unit with the proportion of mentioned papers in the corresponding fields and publication years (the expected values). The family consists of the Equalized Mean-based Normalized Proportion Cited (EMNPC) and the Mean-based Normalized Proportion Cited (MNPC). Hitherto, this new family of indicators has only been studied on rather small samples.

In this study, we investigate the new indicator family empirically on a large scale (multiple complete publication years) and add another member to this family. In statistics, the Mantel–Haenszel (MH) analysis is frequently used for pooling the data from multiple 2 × 2 cross tables based on different subgroups. In this study, we have mentioned and not-mentioned papers of a unit, which have been published in different subject categories and publication years and are compared with the corresponding reference sets. We name the new indicator Mantel–Haenszel quotient (MHq). In the empirical part of this study, we compare the indicator scores with assessments by peers. We are interested whether the indicators can discriminate between different quality levels, which peers assigned to publications. In other words, we investigate the convergent validity of the indicators. The convergent validity can only be tested by using citations, since we can assume that they are related to quality (Diekmann et al. 2012). Thus, the first empirical part is based on citations. In the second part (after confirmation of convergent validity), MHq values are exemplarily presented for Twitter data.

## Indicators for count data with many zeros

The next sections focus on the formulas not only for the calculation of the EMNPC, MNPC, and MHq, but also for the corresponding 95% confidence intervals (CIs). The CI shows the range of possible indicator values: We can be 95% confident that the interval includes the “true” indicator value in the population. Thus, we assume to analyze sample data and infer by using CIs to a larger, inaccessible population (Williams and Bornmann, 2016). Claveau (2016) argues for using inferential statistics with scientometric data as follows: “these observations are realizations of an underlying data generating process … The goal is to learn properties of the data generating process. The set of observations to which we have access, although they are all the actual realizations of the process, do not constitute the set of all possible realizations. In consequence, we face the standard situation of having to infer from an accessible set of observations—what is normally called the sample—to a larger, inaccessible one—the population. Inferential statistics are thus pertinent” (p. 1233).

### Equalized mean-based normalized proportion cited (EMNPC)

Thelwall (2017a, b) proposed the EMNPC as an alternative indicator for count data with many zeros. Here, the proportion of mentioned publications is calculated: suppose that the publication set of a group *g* has *n*_*gf*_ papers in the publication year and subject category combination *f*. *s*_*gf*_ denotes the number of mentioned papers (e.g., on Twitter). *F* denotes all publication year and subject category combinations of the publications in the set. The overall proportion of *g*’s mentioned publications is the number of mentioned publications (*s*_*gf*_) divided by the total number of publications (*n*_*gf*_):

1 $${\raise0.7ex\hbox{${p_{g} = \mathop \sum \nolimits_{f \in F} s_{gf} }$} \!\mathord{\left/ {\vphantom {{p_{g} = \mathop \sum \nolimits_{f \in F} s_{gf} } {\mathop \sum \nolimits_{f \in F} n_{gf} }}}\right.\kern-0pt} \!\lower0.7ex\hbox{${\mathop \sum \nolimits_{f \in F} n_{gf} }$}}$$

However, *p*_*g*_ could lead to misleading results, if the publication set *g* includes many publications which appeared in fields with many mentioned papers. Thus, Thelwall (2017a, b) proposes to artificially treat *g* as having the same number of publications in each year and subject category combination. Thelwall (2017a, b) fixes it to the arithmetic mean of numbers in each combination. However, he recommends excluding combinations of *g* with only a few publications in the analysis. Thus, the equalized sample proportion $\hat{p}_{g}$ is the average of the proportions in each combination:

2 $$\hat{p}_{g} = \frac{{\mathop \sum \nolimits_{f \in F} \frac{{s_{gf} }}{{n_{gf} }}}}{\left[ F \right]}$$

The corresponding equalized sample proportion of the world (*w*, i.e., all papers in the analyzed set) is:

3 $$\hat{p}_{w} = \frac{{\mathop \sum \nolimits_{f \in F} \frac{{s_{wf} }}{{n_{wf} }}}}{\left[ F \right]}$$

In Eqs. (2) and (3), [*F*] is the number of subject category and publication year combinations in which the group (in case of Eq. (2)) and the world (in case of Eq. (3)) publish. The equalized group sample proportion has the following undesirable property: it treats *g* as if the average number of mentioned publications does not vary between the subject categories. The ratio of both equalized sample proportions (group and world) is the EMNPC:

4 $${\text{EMNPC}} = \hat{p}_{g} /\hat{p}_{w}$$

According to Bailey (1987) and Thelwall (2017a), CIs for the EMNPC can be calculated:

5 $${\text{EMNPC}}_{L} = \exp \left( {\ln \left( {\frac{{\hat{p}_{g} }}{{\hat{p}_{w} }}} \right) - 1.96\sqrt {\frac{{(n_{g} - \hat{p}_{g} n_{g} )/(\hat{p}_{g} n_{g} )}}{{n_{g} }} + \frac{{(n_{w} - \hat{p}_{w} n_{w} )/(\hat{p}_{w} n_{w} )}}{{n_{w} }}} } \right)$$

6 $${\text{EMNPC}}_{U} = \exp \left( {\ln \left( {\frac{{\hat{p}_{g} }}{{\hat{p}_{w} }}} \right) + 1.96\sqrt {\frac{{(n_{g} - \hat{p}_{g} n_{g} )/(\hat{p}_{g} n_{g} )}}{{n_{g} }} + \frac{{(n_{w} - \hat{p}_{w} n_{w} )/(\hat{p}_{w} n_{w} )}}{{n_{w} }}} } \right)$$

In Eqs. (5) and (6), *n*_*w*_ is the total sample size of the world, and *n*_*g*_ is the total sample size of group *g*. However, according to Thelwall (2017a), these “confidence intervals seem to be only approximations, however, as they can differ from bootstrapping estimates” (p. 133).

### Mean-based normalized proportion cited (MNPC)

The other indicator proposed by Thelwall (2017a) is the Mean-based Normalized Proportion Cited (MNPC) which is calculated as follows: For each publication which is mentioned at least once (e.g., mentioned on Twitter), the reciprocal of the world proportion mentioned for the corresponding subject category and publication year replaces the number of mentions. All unmentioned publications remain at zero. If *p*_*gf*_ = *s*_*gf*_/*n*_*gf*_ is the proportion of mentioned publications in set *g* in the corresponding subject category and publication year combination *f* and *p*_*wf*_ = *s*_*wf*_/*n*_*wf*_ the proportion of world’s mentioned publications in the same year and subject category combination *f*, then using the number of citations/mentions *c*_*i*_:

7 $$r_{i} = \left\{ {\begin{array}{*{20}l} {0,} \hfill & {{\text{if}}\quad c_{i} = 0} \hfill \\ {1/p_{wf} ,} \hfill & { {\text{if}}\quad c_{i} > 0\quad {\text{where paper}}\;i\;{\text{is from year and subject category combination}}\;f} \hfill \\ \end{array} } \right.$$

The MNPC calculation follows the calculation of the MNCS (Waltman et al. 2011) and is defined as:

8 $${\text{MNPC}} = \frac{{\left( {r_{1} + r_{2} + \cdots + r_{{n_{g} }} } \right)}}{{n_{g} }}$$

Thelwall (2016, 2017a) proposes an approximate CI for the MNPC based on a standard formula from Bailey (1987). The lower limit *L* (MNPC_*gfL*_) and upper limit *U* (MNPC_*gfU*_) for group *g* in year and subject category combination *f* are calculated in the first step:

9 $${\text{MNPC}}_{gfL} = \exp \left( {\ln \left( {\frac{{\hat{p}_{gf} }}{{\hat{p}_{w} f}}} \right) - 1.96\sqrt {\frac{{(n_{gf} - \hat{p}_{gf} n_{gf} )/(\hat{p}_{gf} n_{gf} )}}{{n_{gf} }} + \frac{{(n_{wf} - \hat{p}_{wf} n_{wf} )/(\hat{p}_{wf} n_{wf} )}}{{n_{wf} }}} } \right)$$

10 $${\text{MNPC}}_{gfU} = \exp \left( {\ln \left( {\frac{{\hat{p}_{gf} }}{{\hat{p}_{w} f}}} \right) + 1.96\sqrt {\frac{{(n_{gf} - \hat{p}_{gf} n_{gf} )/(\hat{p}_{gf} n_{gf} )}}{{n_{gf} }} + \frac{{(n_{wf} - \hat{p}_{wf} n_{wf} )/(\hat{p}_{wf} n_{wf} )}}{{n_{wf} }}} } \right)$$

The group-specific lower and upper limits are used to calculate the MNPC CIs in a second step:

11 $${\text{MNPC}}_{L} = {\text{MNPC}} - \mathop \sum \limits_{f \in F} \frac{{n_{gf} }}{{n_{g} }}\left( {\frac{{p_{gf} }}{{p_{wf} }} - {\text{MNPC}}_{gfL} } \right)$$

12 $${\text{MNPC}}_{U} = {\text{MNPC}} + \mathop \sum \limits_{f \in F} \frac{{n_{gf} }}{{n_{g} }}\left( {{\text{MNPC}}_{gfU} - \frac{{p_{gf} }}{{p_{wf} }}} \right)$$

If any of the world proportions are equal to zero, the MNPC cannot be calculated. If any of the group proportions are equal to zero, CIs cannot be calculated. As solutions, either the corresponding subject category and publication year combinations can be removed from the data or a continuity correction of 0.5 can be added to the number of mentioned and not mentioned publications (Thelwall 2017a). We prefer to use the continuity correction. Plackett (1974) recommends this approach for the calculation of odds ratios. However, according to Thelwall (2017a), these “confidence intervals are only approximations, however, and can differ substantially from bootstrapping estimates” (p. 135).

### Mantel–Haenszel quotient (MHq)

The recommended method for pooling the data from multiple 2 × 2 cross tables—based on different subgroups (which are part of a larger population)—is the Mantel–Haenszel (MH) analysis (Hollander and Wolfe 1999; Mantel and Haenszel 1959; Sheskin 2007). According to Fleiss et al. (2003) the method “permits one to estimate the assumed common odds ratio and to test whether the overall degree of association is significant. … The fact that the methods use simple, closed-form formulas has much to recommend it” (p. 250). The results of Radhakrishna (1965) point out that the MH approach is empirically and formally valid against the background of clinical trials.

The MH analysis yields a summary odds ratio for multiple 2 × 2 cross tables. We call this summary odds ratio MHq. If the impact of units in science is compared with reference sets (the world), the 2 × 2 cross tables (which are pooled) consist of the number of publications mentioned and not mentioned in subject category and publication year combinations *f* (see Table 1). Publications of group *g* are part of the publications in the world.

**Table 1:** 2 × 2 subject-specific cross table

	Number of mentioned publications	Number of unmentioned publications
Group *g*	*s*_*gf*_	*n*_*gf*_ − *s*_*gf*_
World	*s*_*wf*_	*n*_*wf*_ − *s*_*wf*_

The MHq calculation starts by defining some auxiliary variables:

13 $$R_{f} = \frac{{s_{gf} \left( {n_{wf} - s_{wf} } \right)}}{{n_{wf} + n_{gf} }}\quad {\text{with}}\quad R = \mathop \sum \limits_{f = 1}^{F} R_{f} ,$$

14 $$S_{f} = \frac{{\left( {n_{gf} - s_{gf} } \right)s_{wf} }}{{n_{wf} + n_{gf} }}\quad {\text{with}}\quad S = \mathop \sum \limits_{f = 1}^{F} S_{f} ,$$

15 $$P_{f} = \frac{{s_{gf} + \left( {n_{wf} - s_{wf} } \right)}}{{n_{wf} + n_{gf} }}\quad {\text{with}}\quad Q_{f} = 1 - P_{f}$$

The MHq is defined as:

16 $${\text{MHq}} = \frac{R}{S}$$

The MHq CIs are calculated as recommended by Fleiss et al. (2003). The variance of ln(MHq) is estimated by:

17 $$\widehat{\text{Var}}\left[ {\ln \left( {\text{MHq}} \right)} \right] = \frac{1}{2}\left\{ {\frac{{\mathop \sum \nolimits_{f = 1}^{F} P_{f} R_{f} }}{{R^{2} }} + \frac{{\mathop \sum \nolimits_{f = 1}^{F} \left( {P_{f} S_{f} + Q_{f} R_{f} } \right)}}{RS} + \frac{{\mathop \sum \nolimits_{f = 1}^{F} Q_{f} S_{f} }}{{S^{2} }}} \right\}$$

Calculation of the MHq CIs is performed as follows:

18 $${\text{MHq}}_{L} = \exp \left[ {\ln \left( {\text{MHq}} \right) - 1.96\sqrt {\widehat{\text{Var}}\left[ {\ln \left( {\text{MHq}} \right)} \right]} } \right]$$

19 $${\text{MHq}}_{U} = \exp \left[ {\ln \left( {\text{MHq}} \right) + 1.96\sqrt {\widehat{\text{Var}}\left[ {\ln \left( {\text{MHq}} \right)} \right]} } \right]$$

It is an advantage of the MHq that the world average has a value of 1. This is similar to the EMNPC and MNPC and simplifies the interpretation. A further advantage of the MHq is that the result can be interpreted as a percentage relative to the world average. MHq = 1.20, e.g., means that the publication set under study has an impact which is 20% above average.

## Data sets used

As data sets, we used publications of the Web of Science (WoS) from our in-house database—derived from the Science Citation Index Expanded (SCI-E), Social Sciences Citation Index (SSCI), and Arts and Humanities Citation Index (AHCI) provided by Clarivate Analytics. All papers of the document type “article” with DOI published between 2010 and 2013 were included in the study. The citation counts are restricted to citations with a three-year citation window—excluding the publication year (Glänzel and Schoepflin 1995). The overlapping WoS subject categories are used for field classification (Rons 2012, 2014). We include only subject categories in this study where (1) more than 9 papers are assigned to and (2) the number of cited and uncited publications is greater than zero. This should avoid statistical and numerical problems. These restrictions resulted in a data set including 4,490,998 publications.

We matched the publication data with peers’ recommendations from F1000Prime via the DOI. F1000Prime is a post-publication recommendation system of publications which have been published mainly in medical and biological journals. A peer-nominated global “Faculty” of leading scientists and clinicians selects and rates the publications and explains their importance. Thus, only a restricted set from the publications in these disciplines covered is reviewed. Most of the publications are actually not. Faculty members can select and evaluate any publication of interest. Faculty members rate the publications as “Recommended,” “Must read”, or “Exceptional”. This is equivalent to recommendation scores (RSs) of 1, 2, or 3, respectively. Since publications can be recommended multiple times, we calculated an average RS (${\overline{\text{FFa}}}$):

20 $${\overline{\text{FFa}}} = \frac{1}{{i_{\max}}}\mathop \sum \limits_{i}^{{i_{\max}}} RS_{i}$$

The papers are categorized depending on their ${\overline{\text{FFa}}}$ value:

- ${\overline{\text{FFa}}} = 0$: publications which are not recommended (Q0)
- $0 < {\overline{\text{FFa}}} \le 1.0$: recommended publications with a rather low average score (Q1):
- ${\overline{\text{FFa}}} > 1.0$: recommended publications with a rather high average score (Q2):

We take these three groups (Q0, Q1, and Q2) as our unit of analysis. Following Waltman and Costas (2014), we only considered subject categories where a publication with an F1000Prime recommendation is assigned to.

Whereas the first empirical part of this study is based on citation counts, the second empirical part focusses on Twitter data. The Twitter data were taken from a data set which the company Altmetric has shared with us. We matched the papers with the Twitter information via the paper’s DOI. Papers which were unknown to Altmetric were treated as papers with zero tweets. With the number of publications and proportion of uncited and untweeted publications, Tables 2 and 3 show overviews of the data included in this study. It is clearly visible in Table 3 that Twitter data possess many zeros, but citation data do not.

**Table 2:** Number of papers included in this study broken down by different sources (citations and Twitter groups), publication year, and ${\overline{\text{FFa}}}$

Years	${\overline{\text{FFa}}}$	Citations	Twitter groups				
			All	Researchers	Science communicators	Practitioners	Members of the public
2010	Q0	628,862	627,082	587,563	505,205	504,052	626,116
	Q1	6576	6630	6286	5941	6189	6622
	Q2	4368	4413	4293	4089	4176	4413
2011	Q0	681,749	683,815	669,966	614,564	598,772	683,815
	Q1	6324	6439	6335	6065	6258	6439
	Q2	4418	4494	4459	4391	4424	4494
2012	Q0	733,813	737,074	730,917	704,025	706,521	736,992
	Q1	5826	5974	5944	5846	5888	5973
	Q2	5042	5176	5163	5133	5134	5176
2013	Q0	785,961	788,706	786,486	772,364	770,555	788,758
	Q1	4176	4254	4249	4208	4233	4255
	Q2	6361	6512	6512	6470	6503	6512

**Table 3:** Proportion of uncited and untweeted papers broken down by different sources (citations and Twitter groups), publication year, and ${\overline{\text{FFa}}}$

Years	${\overline{\text{FFa}}}$	Citations	Twitter groups				
			All	Researchers	Science communicators	Practitioners	Members of the public
2010	Q0	10.36	95.63	98.88	99.43	99.08	96.44
	Q1	0.84	86.50	95.31	98.32	97.24	88.95
	Q2	0.43	76.21	89.89	96.50	94.49	80.04
2011	Q0	10.61	87.99	97.04	98.69	98.19	89.96
	Q1	1.12	69.13	89.11	95.50	93.94	72.71
	Q2	0.68	51.91	76.74	90.21	89.10	56.25
2012	Q0	10.41	72.47	92.03	96.16	95.68	77.23
	Q1	1.08	38.47	75.74	84.76	84.00	44.78
	Q2	0.46	23.59	56.11	72.10	75.94	29.42
2013	Q0	10.84	68.12	89.21	94.33	93.62	73.53
	Q1	1.39	31.29	70.35	81.87	77.79	37.53
	Q2	0.50	21.10	50.58	69.30	69.71	26.87

In most of the previous studies, which are based on Twitter data, counts of Twitter mentions are used. In this study, we differentiate the Twitter data further on and focus on different Twitter groups: researchers, science communicators, practitioners, and members of the public. These groups are defined by Altmetric based on keyword matching (Adie 2016):

- Researcher sample keywords: “Post doc”, “post-doc”, “post-doctoral”, …
- Science communicator sample keywords: “news service”, “Magazine”, …
- Practitioner sample keywords: “medic”, “medical student”, “Pediatrician”, …
- Member of the public is a Twitter user which does not fall into another of the previous three groups.

## Results

### Empirical analysis using citations

It is an established way of analyzing the convergent validity of indicators comparing the indicator values with peer evaluations (Garfield 1979; Kreiman and Maunsell 2011). Convergent validity is defined as the degree to which two measurements of a construct (here: two indicators of scientific quality) with a theoretical relationship are also empirically related. This approach has been justified by Thelwall (2017b) as follows: “if indicators tend to give scores that agree to a large extent with human judgements then it would be reasonable to replace human judgements with them when a decision is not important enough to justify the time necessary for experts to read the articles in question” (p. 4). Several publications studying the relationship between Research Excellence Framework (REF) outcomes and citations reveal considerable relationships in different fields, such as psychology and biological science (Butler and McAllister 2011; Mahdi et al. 2008; McKay 2012; Smith and Eysenck 2002; Traag and Waltman 2017; Wouters et al. 2015). Similar results have been reported for the Italian research assessment exercise: “The correlation strength between peer assessment and bibliometric indicators is statistically significant, although not perfect. Moreover, the strength of the association varies across disciplines, and it also depends on the discipline internal coverage of the used bibliometric database” (Franceschet and Costantini 2011, p. 284). Bornmann (2011) shows in an overview of studies on journal peer review that better recommendations from peers are related to higher citation impact of the corresponding papers.

The correlation between citation impact scores and RS from F1000Prime has already been investigated in other studies. The results of Bornmann (2015) reveal that about 40% of papers with RS = 1 are highly cited papers; for publications with RS = 2 and RS = 3 the percentages are 60 and 73%, respectively. Waltman and Costas (2014) report “a clear correlation between F1000 recommendations and citations” (p. 433). The previous results on F1000Prime might point out, therefore, that citation-based indicators differentiate between the three quality levels. Looking at it the other way round, the validity of new indicators does not seem to be given if they do not differentiate.

**Fig. 1:**
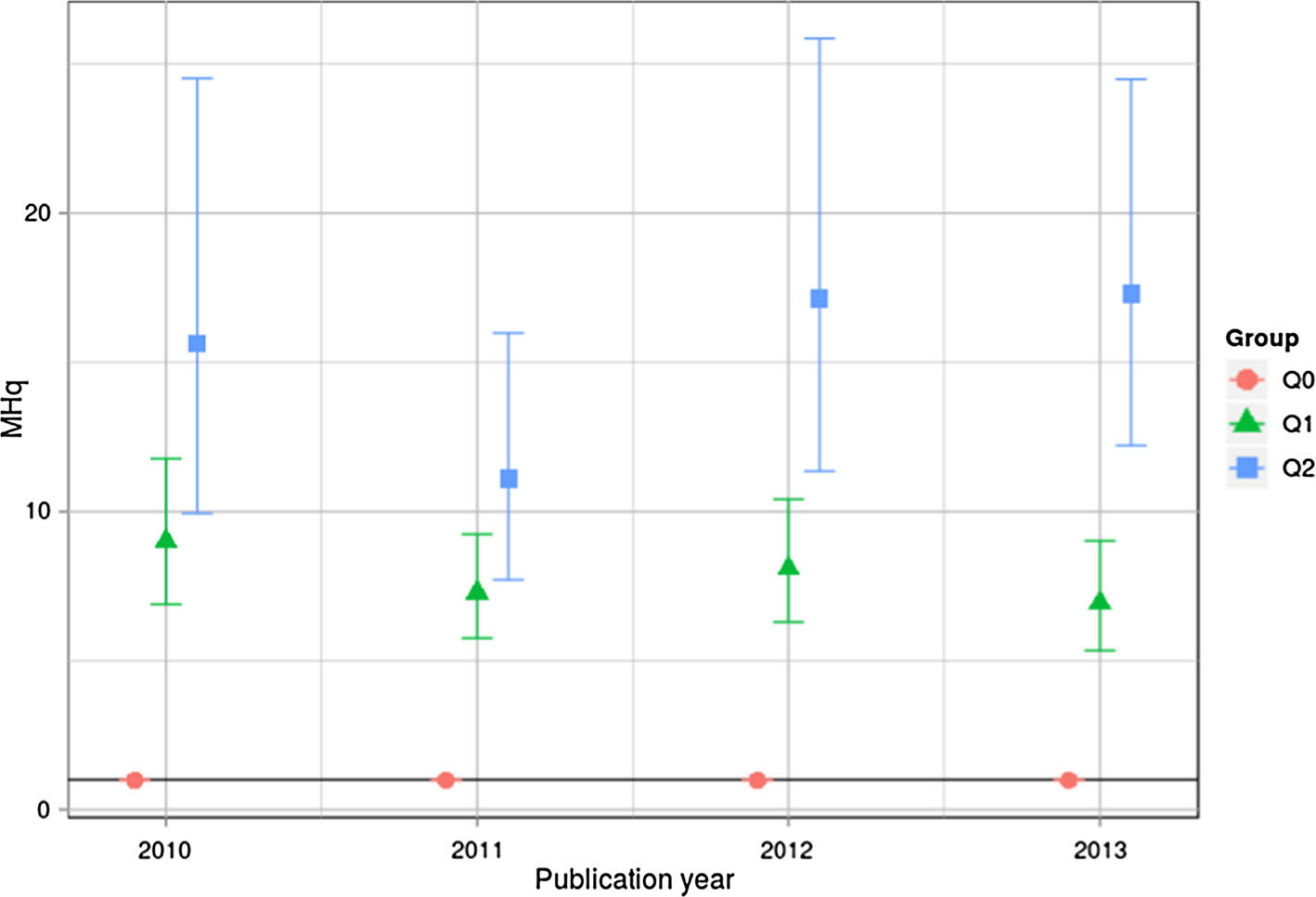
MHqs with CIs for Q0, Q1, and Q2 across four publication years. The horizontal line with MHq = 1 is the worldwide average

Against this backdrop, we analyze in this study the ability of MHq, EMNPC, and MNPC to differentiate between the F1000Prime quality groups. Figure 1 shows the MHqs with CIs for Q0, Q1, and Q2 across four publication years.

The results in Fig. 1 point out that the MHq values are very different for Q0, Q1, and Q2. The average MHq for all years is close to (but below) 1 for Q0. The mean MHq for Q1 is about eight times and that for Q2 is about 15 times higher than the mean MHq for Q0. Thus, the MHq indicator seems to separate significantly between Q0, Q1, and Q2; the MHq values seem to be convergent valid with respect to F1000Prime scores.

**Fig. 2:**
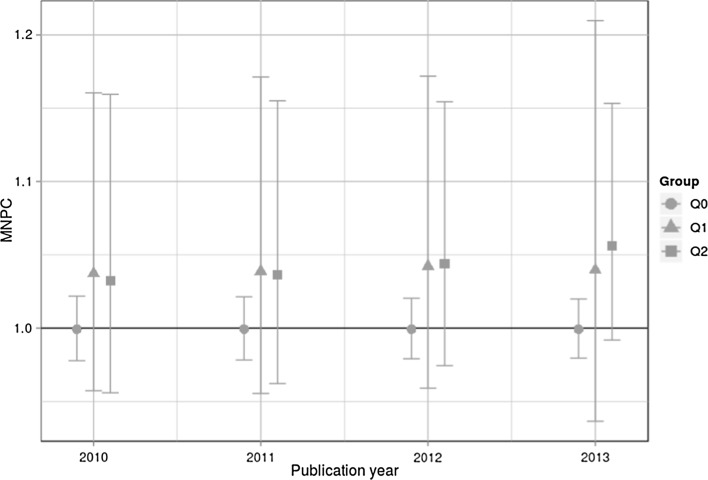
MNPC with CIs for Q0, Q1, and Q2 across four publication years. The horizontal line with MNPC = 1 is the worldwide average

**Fig. 3:**
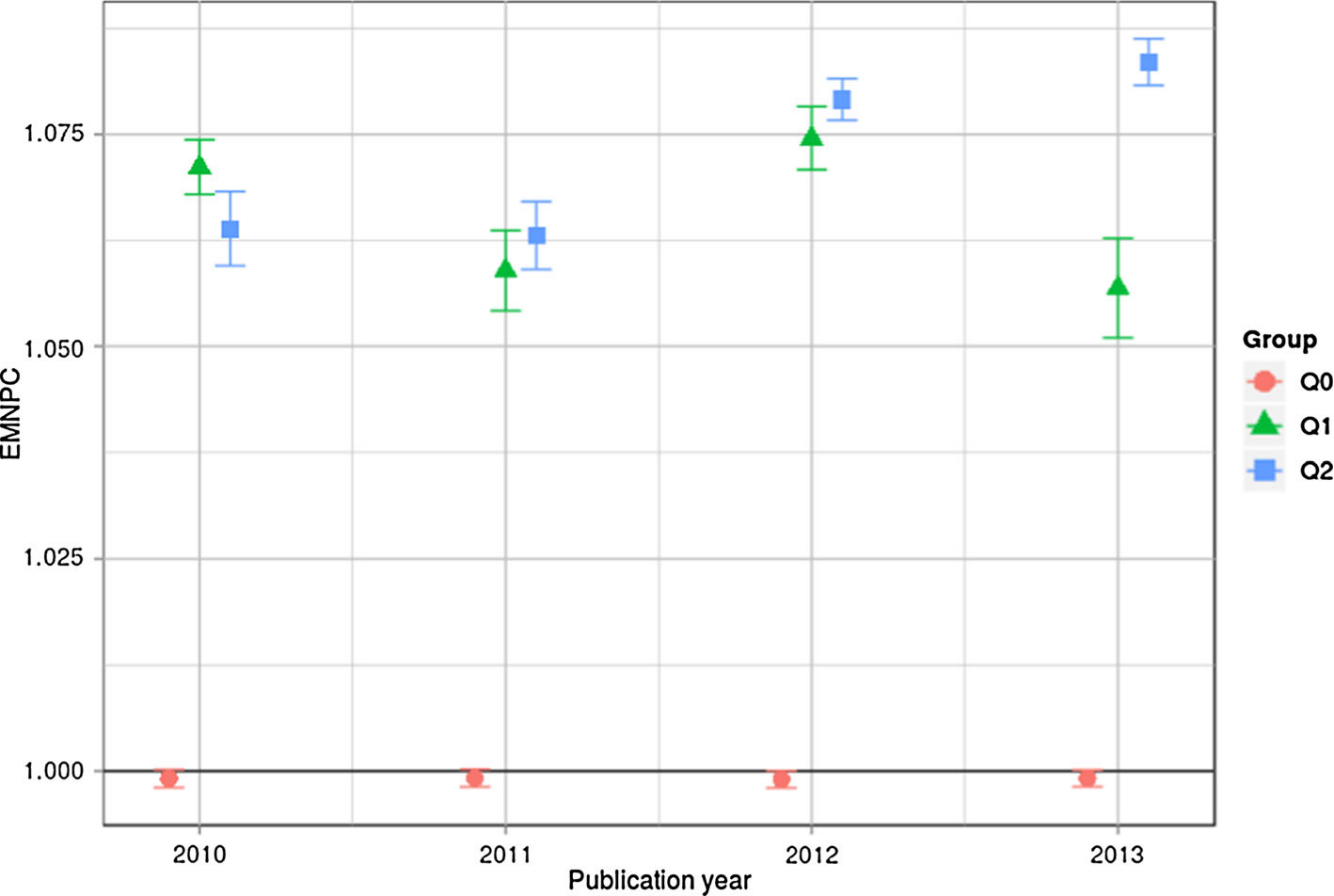
EMNPC with CIs for Q0, Q1, and Q2 across four publication years. The horizontal line with EMNPC = 1 is the worldwide average

The results for MNPC and EMNPC—the two indicators proposed by Thelwall (2017a)—are shown in Figs. 2 and 3. It is clearly visible in both figures that the indicator values for all groups are very close to 1. This result does not accord with Fig. 1 where the MHq values for Q1 and Q2 are significantly greater than 1. Also, as visible in Figs. 2 and 3, the indicator values do not show the expected ordering (analogous to the ordering in Fig. 1: MHq(Q0) < MHq(Q1) < MHq(Q2)) for 2010 in the case of EMNPC and 2010 and 2011 in the case of MNPC. These differences in the results can be interpreted as a first indication that MNPC and EMNPC do not differentiate properly between quality groups as defined by ${\overline{\text{FFa}}}$ values.

### Empirical analysis using Twitter data

**Fig. 4:**
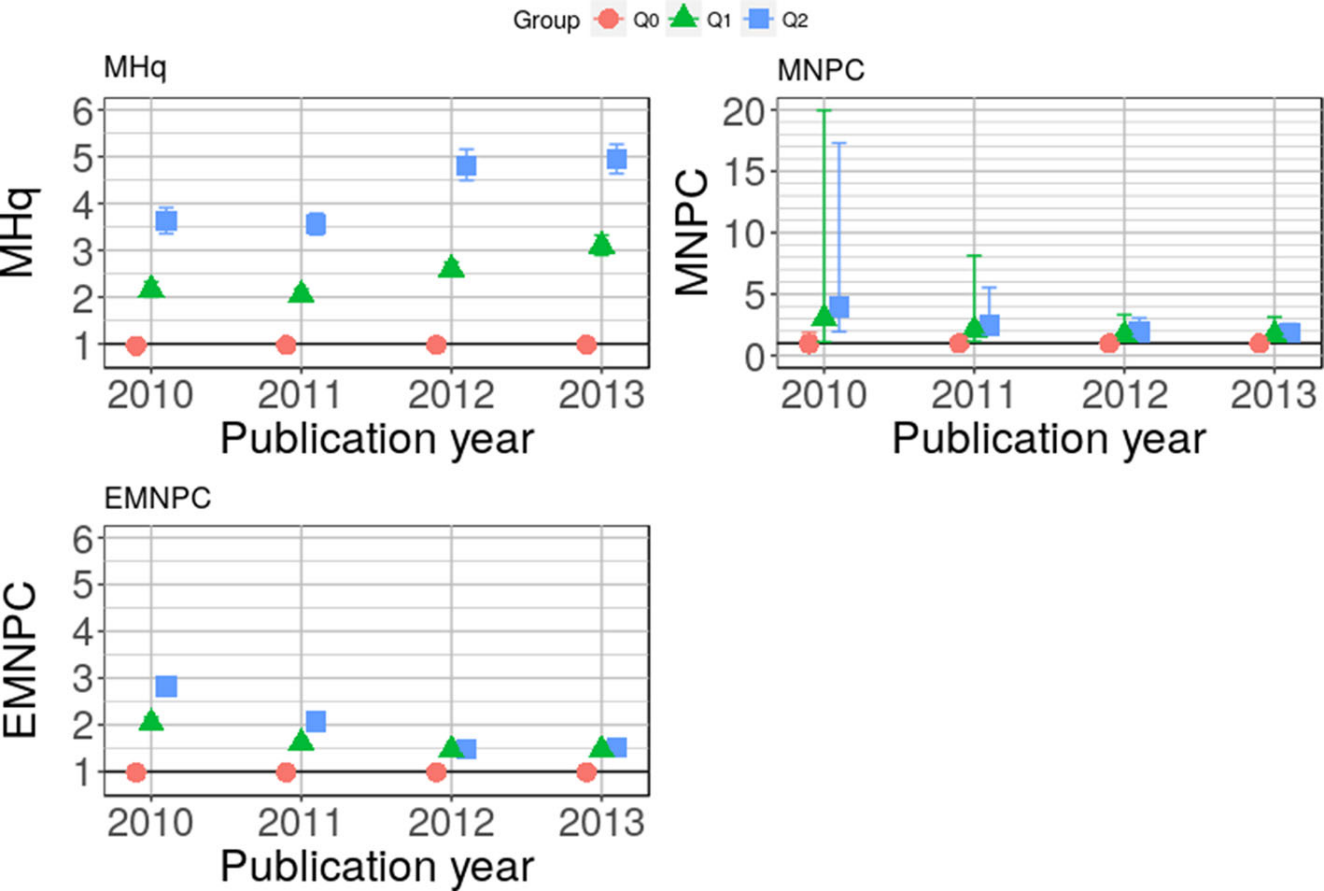
MHq, MNPC, and EMNPC with CIs for Q0, Q1, and Q2 across four publication years for Twitter counts. The horizontal line with indicator values of 1 is the worldwide average

In the previous section, we demonstrated that the MHq is convergent valid using citation data compared with post-publication recommendation scores from F1000Prime. The MHq is able to distinguish between different scientific quality levels as defined by F1000Prime scores. In this section, we start by analyzing the ability of MHq, MNPC, and EMNPC to discriminate between the quality levels Q0, Q1, and Q2 for Twitter data in general. Figure 4 shows the three indicator’s performances for Twitter counts. Only the MHq can distinguish between the quality levels for all publication years in the case of Twitter data. The CIs for EMNPC are overlapping for the publication years 2012 and 2013. In the case of MNPC, the CIs are overlapping for all publication years. In general, our observations from citation counts are substantiated by the analysis of Twitter counts.

**Fig. 5:**
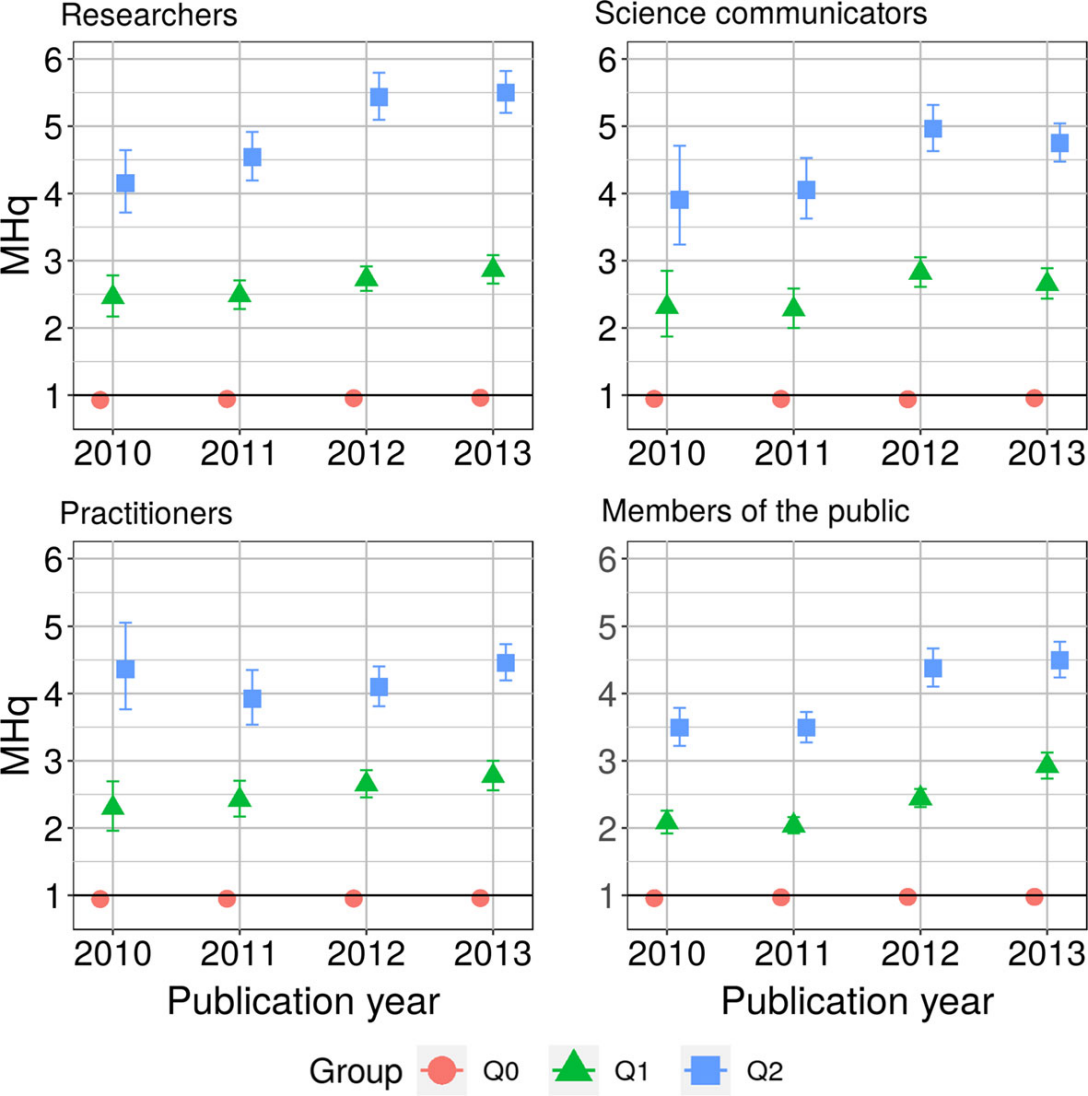
MHq values for Q0, Q1, and Q2 with CIs differentiated by Twitter groups and publication years. The horizontal line with MHq = 1 is the worldwide average

Next, we determine whether and to which extent different Twitter groups (as defined by the company Altmetric) can distinguish between the same quality levels. Figure 5 shows the MHq results for researchers, science communicators, practitioners, and members of the public.

All four Twitter groups show the expected ordering with MHq(Q0) < MHq(Q1) < MHq(Q2). For all four Twitter groups, the quality group Q0 is close to but below 1. The MHq values for the quality groups Q1 and Q2 are between 2 and 4 and between 4 and 6, respectively. Compared to citation data, all four Twitter groups show a much weaker association to scientific quality than citations, by a factor of about three. In Fig. 1, which shows the results for citation data, MHq(Q1) is between 7 and 9, and MHq(Q2) is between 11 and 18.

The results in Fig. 5 further reveal that the association to scientific quality is on a similar level for all four Twitter groups. Since researchers should be able to assess scientific quality better than the other groups in the figure, the association to quality is somewhat stronger for researchers in the figure than for the other groups. This can be seen from the somewhat higher MHq values for researchers (e.g., 5.5 in 2013) in comparison with the other groups (i.e., 4.5 for practitioners, 4.7 for science communicators, and 4.5 for members of the public in 2013).

## Discussion

Much of the altmetrics data is sparse (Neylon 2014). A metric based on many zero values is not informative for research evaluation purposes in the first place (Thelwall et al. 2016). Thelwall (2017a, b) introduced a new family of field- and time normalized indicators for sparse data including EMNPC and MNPC. Here, the proportion of mentioned publications of a unit (e.g., a researcher) is compared with the expected values (the proportion of mentioned publications in the corresponding publication years and fields).

EMNPC and MNPC differ from most of the indicators used in bibliometrics and altmetrics. Usually, an indicator value is calculated for each publication. The publication-based indicator values can then be aggregated by the user, for example, by averaging or summing. Instead, the indicators of the new family are based on the calculation of the indicator values for publication sets of groups (e.g., universities). This property implies that the new indicators cannot be used as versatilely as the usual bibliometric (and altmetric) indicators. However, they are able (by construction) to handle data with many zeros properly which the usual indicators do not.

In this study, we added a further variant to the family—the MHq—and analyzed all three variants empirically. We started by analyzing the convergent validity of the indicators based on citation data. Citation data can be used to formulate predictions, which can be empirically validated. Thus, we studied whether EMNPC, MNPC, and MHq are able to validly differentiate between three different quality levels—as defined by RS from F1000 (${\overline{\text{FFa}}}$). By comparing the indicator values with peer recommendations, we were able to test whether the indicators discriminate between different quality levels. We also analyzed the ability of the three indicators to validly differentiate the quality levels on the basis of Twitter counts.

The results point out that EMNPC and MNPC cannot validly discriminate between different quality levels as defined by peers. The EMNPC and MNPC values are close to the worldwide average—independent of the quality levels. The CIs substantially overlap in many comparisons. Thus, the convergent validity of the EMNPC and MNPC does not seem to be given. In contrast to these indicators, the MHq is able to discriminate between the quality levels.

Because of the positive results for the MHq, we applied the MHq to Twitter counts of four different groups as defined by the company Altmetric: researchers, science communicators, practitioners, and members of the public. If Twitter counts are intended to be used for research evaluation, a substantial relationship to scientific quality should be given. Otherwise, Twitter counts should not be employed in research evaluation. Our investigation of MHq values based on Twitter data reveals a weak relationship between Twitter counts and scientific quality. This relationship is much weaker than that between citation counts and scientific quality.

Our study of the relationship of different Twitter groups’ data to scientific quality is directed at specific societal groups. Earlier, we studied the directed societal impact measurement for different status groups of Mendeley data (Bornmann and Haunschild 2017). Researchers on Twitter show a slightly stronger relationship to scientific quality than other societal groups, but the differences are only minor.

This study follows the important initiative of Thelwall (2017a, b) to develop new indicators for data with many zeros. The current study is the first independent attempt to investigate the new indicator family empirically. This family is important for altmetrics data. Thus, we need further studies focusing on various sources with sparse data (in addition to Twitter). Since F1000 concentrates on biomedicine, future empirical studies should analyze the new family in other disciplines.

The use of F1000Prime peer evaluations as a measure of quality may be a limitation of our study. Peers might be biased from citation counts of the assessed papers or the Twitter accounts they follow. Thus, the recommendation of a paper might be the result of a tweet mentioning the paper. However, F1000Prime faculty members usually recommend papers early after publications so that at least citation rates should not shape their view of many recommended papers.

## References

[CR1] Adie, E. (2016). Personal Communication. Email correspondence on 18 January 2016.

[CR2] Bailey BJR (1987). Confidence-limits to the risk ratio. Biometrics.

[CR3] Bornmann L (2011). Scientific peer review. Annual Review of Information Science and Technology.

[CR4] Bornmann L (2014). Validity of altmetrics data for measuring societal impact: A study using data from Altmetric and F1000Prime. Journal of Informetrics.

[CR5] Bornmann L (2015). Inter-rater reliability and convergent validity of F1000Prime peer review. Journal of the Association for Information Science and Technology.

[CR6] Bornmann L, Haunschild R (2016). How to normalize Twitter counts? A first attempt based on journals in the Twitter Index. Scientometrics.

[CR7] Bornmann L, Haunschild R (2016). Normalization of Mendeley reader impact on the reader- and paper-side: A comparison of the mean discipline normalized reader score (MDNRS) with the mean normalized reader score (MNRS) and bare reader counts. Journal of Informetrics.

[CR8] Bornmann L, Haunschild R (2017). Measuring field-normalized impact of papers on specific societal groups: An altmetrics study based on Mendeley Data. Research Evaluation.

[CR9] Butler L, McAllister I (2011). Evaluating university research performance using metrics. European Political Science.

[CR10] Claveau F (2016). There should not be any mystery: A comment on sampling issues in bibliometrics. Journal of Informetrics.

[CR11] Diekmann A, Naf M, Schubiger M (2012). The impact of (Thyssen)-awarded articles in the scientific community. Kölner Zeitschrift für Soziologie und Sozialpsychologie.

[CR12] Erdt M, Nagarajan A, Sin S-CJ, Theng Y-L (2016). Altmetrics: An analysis of the state-of-the-art in measuring research impact on social media. Scientometrics.

[CR13] Fairclough R, Thelwall M (2015). National research impact indicators from Mendeley readers. Journal of Informetrics.

[CR14] Fleiss J, Levin B, Paik MC (2003). Statistical methods for rates and proportions.

[CR15] Franceschet M, Costantini A (2011). The first Italian research assessment exercise: A bibliometric perspective. Journal of Informetrics.

[CR16] Galloway LM, Pease JL, Rauh AE (2013). Introduction to altmetrics for science, technology, engineering, and mathematics (STEM) librarians. Science & Technology Libraries.

[CR17] Garfield E (1979). Citation indexing: Its theory and application in science, technology, and humanities.

[CR18] Glänzel W, Schoepflin U (1995). A bibliometric study on aging and reception processes of scientific literature. Journal of Information Science.

[CR19] Haunschild R, Bornmann L (2016). Normalization of Mendeley reader counts for impact assessment. Journal of Informetrics.

[CR20] Haunschild, R., & Bornmann, L. (2017). Normalization of zero-inflated data: An empirical analysis of a new indicator family. In *Proceedings of ISSI 2017: The 16th International Conference on Scientometrics and Informetrics* (pp. 448–459). China: Wuhan University.

[CR21] Haunschild R, Schier H, Bornmann L (2016). Proposal of a minimum constraint for indicators based on means or averages. Journal of Informetrics.

[CR22] Haustein S (2016). Grand challenges in altmetrics: Heterogeneity, data quality and dependencies. Scientometrics.

[CR23] Haustein S, Larivière V, Thelwall M, Amyot D, Peters I (2014). Tweets vs. Mendeley readers: How do these two social media metrics differ?. IT-Information Technology.

[CR24] Hollander M, Wolfe DA (1999). Nonparametric statistical methods.

[CR25] Kreiman G, Maunsell JHR (2011). Nine criteria for a measure of scientific output. Frontiers in Computational Neuroscience.

[CR26] Mahdi S, d’Este P, Neely AD (2008). Citation counts: Are they good predictors of RAE scores? A bibliometric analysis of RAE 2001.

[CR27] Mantel N, Haenszel W (1959). Statistical aspects of the analysis of data from retrospective studies of disease. Journal of the National Cancer Institute.

[CR28] McKay S (2012). Social policy excellence–peer review or metrics? Analyzing the 2008 research assessment exercise in social work and social policy and administration. Social Policy & Administration.

[CR29] National Information Standards Organization. (2016). *Outputs of the NISO alternative assessment metrics project*. Baltimore: National Information Standards Organization (NISO).

[CR30] Neylon, C. (2014). *Altmetrics: What are they good for?* Retrieved from http://blogs.plos.org/opens/2014/10/03/altmetrics-what-are-they-good-for/#.VC8WETI0JAM.twitter. Accessed 6 Oct 2014.

[CR31] Plackett RL (1974). The analysis of categorical data.

[CR32] Radhakrishna S (1965). Combination of results from several 2 × 2 contingency tables. Biometrics.

[CR33] Rons N (2012). Partition-based field normalization: An approach to highly specialized publication records. Journal of Informetrics.

[CR34] Rons, N. (2014). Investigation of partition cells as a structural basis suitable for assessments of individual scientists. In P. Wouters (Ed.), *Proceedings of the science and technology indicators conference 2014 Leiden “Context Counts: Pathways to Master Big and Little Data”* (pp. 463–472). Leider: University of Leiden.

[CR35] Sheskin D (2007). Handbook of parametric and nonparametric statistical procedures.

[CR36] Smith A, Eysenck M (2002). The correlation between RAE ratings and citation counts in psychology.

[CR37] Thelwall, M. (2016). Three practical field normalised alternative indicator formulae for research evaluation. Retrieved from https://arxiv.org/abs/1612.01431.

[CR38] Thelwall M (2017). Three practical field normalised alternative indicator formulae for research evaluation. Journal of Informetrics.

[CR39] Thelwall M (2017). Web indicators for research evaluation: A practical guide.

[CR40] Thelwall M, Kousha K, Dinsmore A, Dolby K (2016). Alternative metric indicators for funding scheme evaluations. Aslib Journal of Information Management.

[CR41] Traag, V. A., & Waltman, L. (2017). Replacing peer review by metrics in the UK REF? In *Paper presented at the ISSI 2016 Wuhan: 16th international society of scientometrics and informetrics conference, Wuhan, China*.

[CR42] Waltman L, Costas R (2014). F1000 Recommendations as a potential new data source for research evaluation: A comparison with citations. Journal of the Association for Information Science and Technology.

[CR43] Waltman L, van Eck NJ, van Leeuwen TN, Visser MS, van Raan AFJ (2011). Towards a new crown indicator: An empirical analysis. Scientometrics.

[CR44] Williams R, Bornmann L (2016). Sampling issues in bibliometric analysis. Journal of Informetrics.

[CR45] Work, S., Haustein, S., Bowman, T. D., & Larivière, V. (2015). *Social media in scholarly communication. A review of the literature and empirical analysis of Twitter use by SSHRC doctoral award recipients*. Montreal: Canada Research Chair on the Transformations of Scholarly Communication, University of Montreal.

[CR46] Wouters, P., Thelwall, M., Kousha, K., Waltman, L., de Rijcke, S., Rushforth, A., & Franssen, T. (2015). *The metric tide: Correlation analysis of REF2014 scores and metrics (Supplementary Report II to the Independent Review of the Role of Metrics in Research Assessment and Management)*. London: Higher Education Funding Council for England (HEFCE).

